# Promoter hypomethylation of NY-ESO-1, association with clinicopathological features and PD-L1 expression in non-small cell lung cancer

**DOI:** 10.18632/oncotarget.18198

**Published:** 2017-05-23

**Authors:** Anderly C. Chüeh, Mun-Sem Liew, Prudence A. Russell, Marzena Walkiewicz, Aparna Jayachandran, Maud H.W. Starmans, Paul C. Boutros, Gavin Wright, Stephen A Barnett, John M. Mariadason, Thomas John

**Affiliations:** ^1^ Ludwig Institute of Cancer Research, Melbourne-Austin Branch, Victoria, Australia; ^2^ Department of Medicine, Austin Health, University of Melbourne, Victoria, Australia; ^3^ Olivia Newton-John Cancer Research Institute, Victoria, Australia; ^4^ Department of Anatomical Pathology, St Vincent’s Hospital, Victoria, Australia; ^5^ School of Cancer Medicine, La Trobe University, Victoria, Australia; ^6^ Informatics and Biocomputing Program, Ontario Institute for Cancer Research, Toronto, Canada; ^7^ Department of Medical Biophysics, University of Toronto, Toronto, Canada; ^8^ Department of Pharmacology & Toxicology, University of Toronto, Toronto, Canada; ^9^ Department of Thoracic Oncology, St Vincent’s Hospital, Victoria, Australia; ^10^ Department of Thoracic Surgery, Austin Hospital, Melbourne, Victoria, Australia

**Keywords:** NY-ESO-1, promoter methylation, PD-L1, biomarker, lung cancer

## Abstract

Cancer-Testis antigens (CTA) are immunogenic molecules with normal tissue expression restricted to testes but with aberrant expression in up to 30% of non-small cell lung cancers (NSCLCs). Regulation of CTA expression is mediated in part through promoter DNA methylation. Recently, immunotherapy has altered treatment paradigms in NSCLC. Given its immunogenicity and ability to be re-expressed through demethylation, NY-ESO-1 promoter methylation, protein expression and its association with programmed death receptor ligand-1 (PD-L1) expression and clinicopathological features were investigated. Lung cancer cell line demethylation resulting from 5-Aza-2′-deoxycytidine treatment was associated with both NY-ESO-1 and PD-L1 re-expression *in vitro* but not increased chemosensitivity. NY-ESO-1 hypomethylation was observed in 15/94 (16%) of patient samples and associated with positive protein expression (*P* < 0.0001). In contrast, PD-L1 expression was observed in 50/91 (55%) but strong expression in only 12/91 (13%) cases. There was no association between NY-ESO-1 and PD-L1 expression, despite resultant re-expression of both by 5-Aza-2′-deoxycytidine. Importantly, NY-ESO-1 hypomethylation was found to be an independent marker of poor prognosis in patients not treated with chemotherapy (HR 3.59, *P* = 0.003) in multivariate analysis. In patients treated with chemotherapy there were no differences in survival associated with NY-ESO-1 hypomethylation. Collectively, these results provided supporting evidence for the potential use of NY-ESO-1 hypomethylation as a prognostic biomarker in stage 3 NSCLCs. In addition, these data highlight the potential to incorporate demethylating agents to enhance immune activation, in tumours currently devoid of immune infiltrates and expression of immune checkpoint genes.

## INTRODUCTION

Lung cancer accounts for most cancer deaths worldwide, with the incidence in the developing world set to rise [1]. While immune checkpoint therapy has already altered treatment paradigms [2–4], lung cancer immunotherapy using vaccination strategies and immunomodulatory agents [5] have largely yielded disappointing results [6]. Responses to single agent Programmed Death 1 (PD-1) and its ligand PD-L1 are modest [∼24%] and combination strategies with chemotherapy and other immunotherapies are the subject of active study [7].

One group of potential targets are the cancer-testis antigens (CTA or CT antigens), which are exclusively expressed in normal testes and placenta but also aberrantly expressed in some tumours. New York-Esophageal-1 (NY-ESO-1), is a CTA whose expression occurs in several cancers including ∼30% lung cancers [8, 9], as well as in ovarian, head and neck cancers, melanoma, sarcomas and neuroblastoma [10]. These antigens are attractive because they are not expressed in somatic tissues and therefore can be used as targets for vaccination and adoptive T cell transfer [11, 12].

There is increasing evidence supporting the role of epigenetic aberrations in early malignant transformation, raising the possibility of identifying novel epigenetic-related chemopreventive strategies or reliable diagnostic tools using epigenetic biomarkers [13]. Promoter DNA methylation is a covalent chemical modification resulting in the addition of a methyl group to the carbon-5-position of cytosine (C) residues that are followed immediately by guanine (G). It has gained recognition as a key molecular mechanism in the regulation of both oncogenes and tumour suppressors. A number of recent reports have indicated that promoter methylation plays a critical role in ensuring that CT antigens located on the X chromosome [10] (CT-X, which accounts of half of all known CT antigens) are silenced in somatic tissues. Demethylating agents such as 5-Aza-2′-deoxycytidine (5-Aza-dC) have been shown to induce type-1 interferon pathways and to upregulate CTA expression [14], suggesting that concomitant program death-1 (PD-1) pathway inhibition may enhance the immune effects of epigenetic modulators on T cells.

We recently reported that NY-ESO-1 protein expression was associated with poorer survival in lung cancer patients but yet increased benefit in patients undergoing cisplatin-based chemotherapy [15]. Herein, we sought to determine whether NY-ESO-1 expression was regulated by promoter methylation in lung tumours, and if so, whether methylation status can be used as a predictive and/or prognostic marker in NSCLC. Furthermore, we investigated whether promoter demethylation using, 5-Aza-dC, a DNA methyltransferase inhibitor, influenced the chemosensitivity of lung cancer cell lines *in vitro*. Lastly, we investigated the effects of demethylation on PD-L1 expression *in vitro* and correlated expression of PD-L1 with NY-ESO-1 expression and methylation in patients with stage 3 NSCLC.

## RESULTS

### NY-ESO-1 promoter is hypermethylated in lung cancer cell lines and the methylation level is predictive of NY-ESO-1 mRNA and protein levels

**Figure 1:**
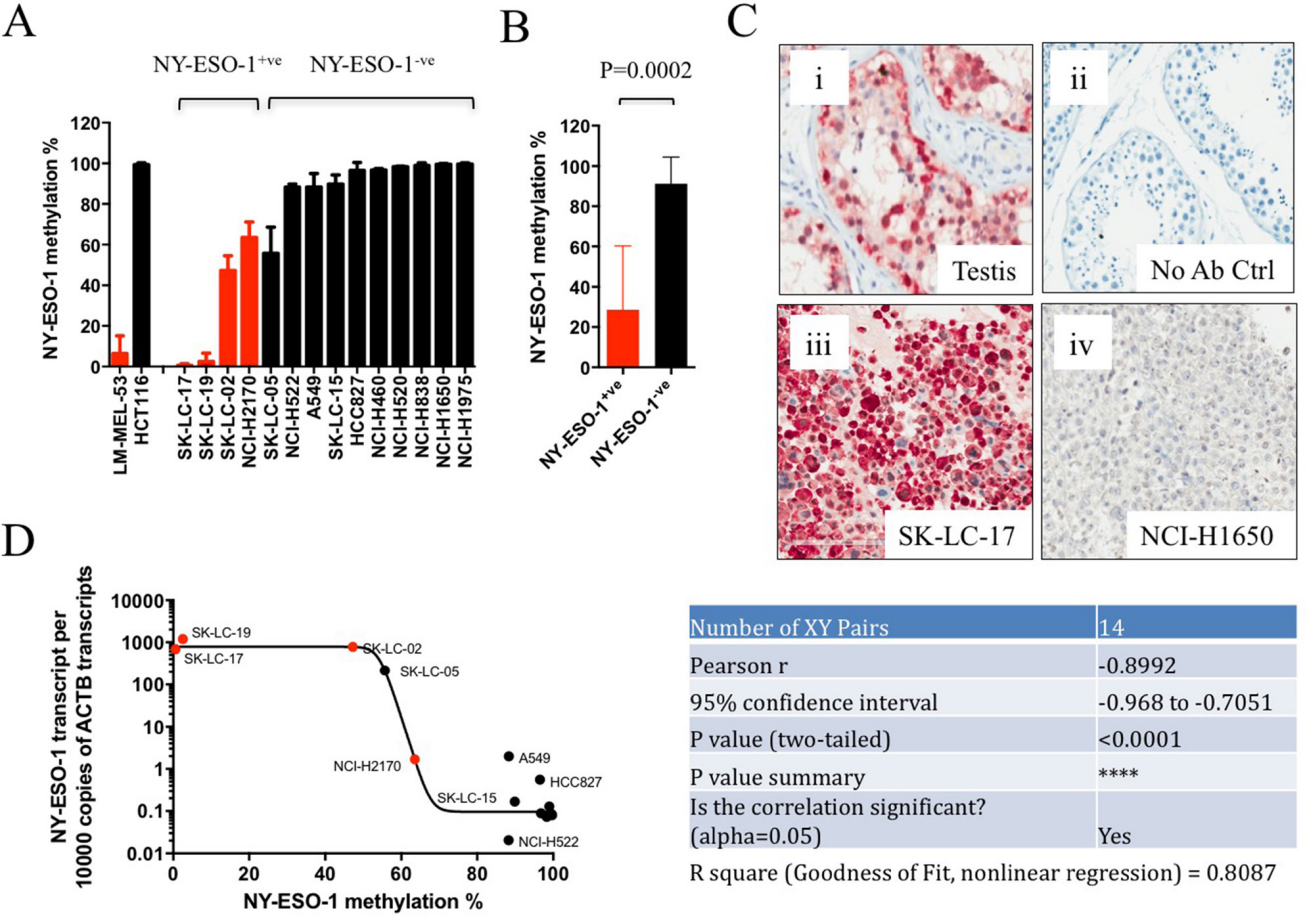
NY-ESO-1 promoter methylation correlated with NY-ESO-1 mRNA and protein expression in lung cancer cell lines (**A**) Bar graph showing the results obtained from qMS-PCR analysis in a panel of 14 lung cancer cell lines. Red bars indicate cell lines stained positive for NY-ESO-1 expression by immunohistochemistry. Data shown are mean % methylation with SD (from 3 or more independent experiments). (**B**) The mean NY-ESO-1 methylation level (% methylation) in NY-ESO-1^+ve^ lung cancer cell lines are significantly lower (i.e. hypomethylated) than NY-ESO-1 negative lung cancer cell lines (*P* < 0.001, Student’s *t*-test). Error bars represent SEM. (**C**) Representative IHC images for NY-ESO-1 using specific anti-NY-ESO-1 antibody (E978) in (i) testis, positive control; (ii) normal lung tissue without primary E978 antibody, negative control; (iii) lung cancer cell line SK-LC-17 and (iv) lung cancer cell line A549. (**D**) Non-linear regression analysis (Pearson correlation, r = –0.8992, *P* < 0.0001) for NY-ESO-1 mRNA expression and promoter DNA methylation (% methylation) in 14 lung cancer cell lines. Cell lines showed positive or negative IHC staining for NY-ESO-1 protein expression were indicated by red and black circles, respectively.

We determined the NY-ESO-1 promoter methylation status in 14 lung cancer cell lines. Variable levels of promoter methylation were observed, with 9 out of 14 cell lines demonstrating >90% NY-ESO-1 hypermethylation (Figure 1A). Three lung cancer cell lines (NCI-2170, SK-LC-02, and SK-LC-05) exhibited approximately equal proportions of methylated and unmethylated NY-ESO-1 alleles (40–60% methylation), while two remaining cell lines (SK-LC-17 and SK-LC19) exhibited NY-ESO-1 hypomethylation, <10% methylation (Figure 1A).

The protein expression level of NY-ESO-1 in all 14 lung cancer cell lines was subsequently investigated using a specific anti-NY-ESO-1 antibody (clone E978) by IHC analysis [16] (Figure 1B, 1C). A NY-ESO-1^+ve^ melanoma cell line, LM-MEL-53, and a NY-ESO-1^–ve^ colorectal cancer cell line, HCT116, were also included in the IHC analysis as positive and negative controls [17]. Among the lung cancer cell lines, 4 out of 14, (SK-LC-17, SK-LC-19, SK-LC-02, and NCI-H2170) stained positive for NY-ESO-1 (Figure 1A, Supplementary Figure 3 and Supplementary Table 3). The NY-ESO-1^+ve^ lung cancer cell lines were found to have completely or partially hypomethylated NY-ESO-1 promoters. In contrast, the NY-ESO-1^-ve^ lines contained hypermethylated NY-ESO-1 promoters, with great majority (9/10) displaying more than 90% methylation (Figure 1A). The mean promoter methylation level was significantly higher (*P* < 0.001, Student’s *t*-test) in the NY-ESO-1^-ve^ cell lines, as compared to NY-ESO-1^+ve^ cell lines (Figure 1B). Importantly, NY-ESO-1 promoter methylation status negatively correlated with NY-ESO-1 mRNA expression (Pearson’s correlation, r = –0.8992, *P* < 0.0001) (Figure 1D). Together, these data confirm a significant inverse correlation between NY-ESO-1 promoter methylation and NY-ESO-1 expression at both the mRNA and protein levels in lung cancer cell lines.

### 5-Aza-2’-deoxycytidine treatment induces NY-ESO-1 and PD-L1 re-expression in NY-ESO-1 methylated lung cancer cell lines

**Figure 2:**
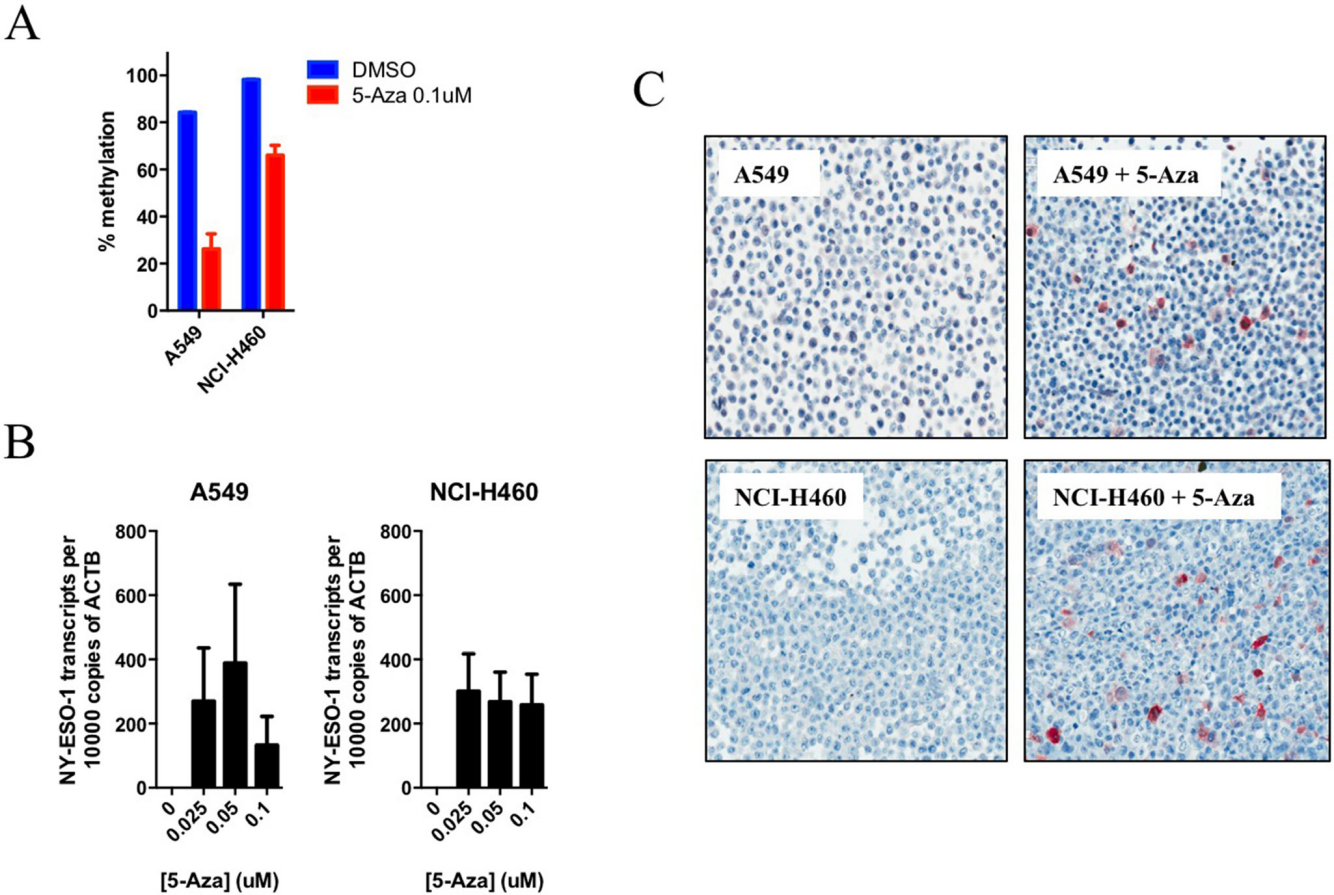
Treatment of lung cancer cells with demethylating agent, 5-Aza-dC, results in the demethylation of the NY-ESO-1 promoter and consequential mRNA and protein re-expression NY-ESO-1 hypermethylated lung cancer cell lines A549 and NCI-H460 were treated with or without 5-Aza-dC for 72 hr at the indicated doses. Results obtained from (**A**) qMS-PCR (*N* = 3, mean with SD), (**B**) qRT-PCR (*N* = 2, mean with SD, perform in triplicates) or (**C**) IHC analysis (representative image) were shown.

Next, we treated a panel of 10 NY-ESO-1^-ve^ cell lines (9 of which showed > 90% NY-ESO-1 methylation) with the demethylating agent 5-Aza-2’-deoxycytidine (5-Aza-dC) for 3 days. The NY-ESO-1 hypomethylated cell lines SK-LC-17 and SK-LC-19 were also treated as additional controls. 5-Aza-dC treatment resulted in a significant reduction of NY-ESO-1 promoter methylation and consequential increased mRNA expression in A549 and NCI-H460 cells (Figure 2A, 2B), but not in SK-LC-17 and SK-LC-19 at similar doses (Supplementary Figures 4 and 5, NY-ESO-1 mRNA expression was already high prior to 5-Aza-dC treatment). In the 10 NY-ESO-1^-ve^ cell lines, treatment resulted in strong IHC expression of NY-ESO-1 in three and patchy positivity in a further three cell lines (Figure 2C, Supplementary Table 3 and Supplementary Figure 6).

Similarly, *de novo* PD-L1 expression was observed in 2/10 cell lines, but on exposure to 5-Aza-dC the positive cell lines remained positive but a further six (out of eight) previously negative cell lines became positive. There was no association between PD-L1 re-expression and NY-ESO-1 re-expression (Supplementary Table 3 and Supplementary Figure 7).

### NY-ESO-1 promoter hypomethylation is a prognostic marker in NSCLC

**Figure 3:**
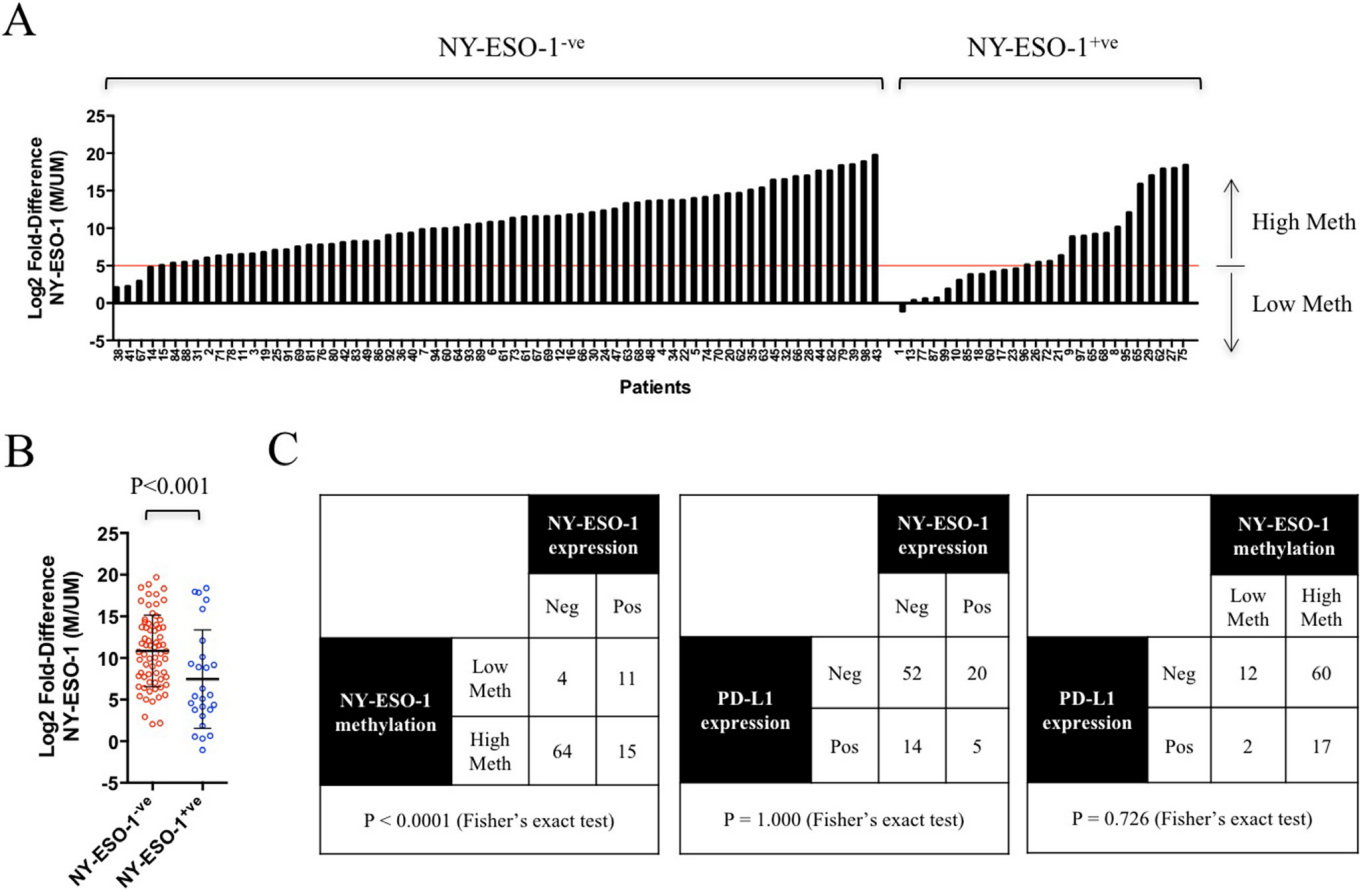
NY-ESO-1 promoter methylation correlated with NY-ESO-1 protein expression in lung cancer patient samples (**A**) NY-ESO-1 promoter methylation in 94 human FFPE lung cancer samples, grouped into NY-ESO-1 negative and positive cohorts. (**B**) Significant differential methylation (*P* < 0.001, Student’s *t*-test) was observed between NY-ESO-1 positive and negative groups. Error bars represent SD. (**C**) NY-ESO-1 promoter hypomethylation was associated with NY-ESO-1 protein expression, while NY-ESO-1 hypermethylation was associated with lack of detectable NY-ESO-1 expression in IHC analysis. A significant association between NY-ESO-1 hypomethylation and protein expression was found, as determined by Fisher’s exact test (*P* < 0.0001). No associated between PD-L1 expression and NY-ESO-1 expression or methylation was observed.

To investigate whether the promoter methylation status of NY-ESO-1 can serve as a prognostic marker, we performed qMS-PCR analysis in a cohort of 99 NSCLC tumours using DNA extracted from FFPE blocks. Matching NY-ESO-1 expression was determined by IHC analysis in these tumours (Figure 3A and Supplementary Figure 8). qMS-PCR analysis was unsuccessful for 5 tumour specimens due to the poor quality of DNA obtained from FFPE samples. 64/68 (94%) NY-ESO-1^-ve^ tumours were hypermethylated, while 11/26 (42%) NY-ESO-1^+ve^ tumours were hypomethylated (Figure 3A and Supplementary Figure 8). Importantly, a significant association between NY-ESO-1 promoter hypomethylation and NY-ESO-1 protein expression (*P* < 0.0001) was found in this lung cancer cohort (Figure 3B, 3C). There were no associations between NY-ESO-1 promoter hypomethylation and clinicopathological features, such as the presence of specific oncogenic mutations, tumour histology or stage (Table 1). Despite not reaching statistical significance, there is a larger number of adenocarcinoma exhibiting NY-ESO-1 promoter hypermethylation, when compared to that of squamous cell carcinoma. No significant association was found between PD-L1 positivity and NY-ESO-1 promoter methylation status or NY-ESO-1 expression (Figure 3C).

**Table 1:** Clinicopathological features associated with NY-ESO-1 promoter methylation in stage 3 NSCLC patients

Variables	NY-ESO-1 Low Meth *N* = 14 n (%)	NY-ESO-1 High Meth *N* = 80 n (%)	*P*-value
Age, mean (range) years	63.1 (46.2 – 85.6)	64.4 (29.0 – 81.4)	0.656
Male	9 (64)	43 (54)	0.566
Smoker/Ex-Smoker	14 (100)	66 (83)	0.119
Histology Adenocarcinoma Squamous Other	4 (29) 5 (36) 5 (36)	47 (59) 22 (28) 11 (14)	0.059
Mutations TP53 KRAS EGFR PIK3CA MET	2 (14) 2 (14) 0 (0) 1 (7) 0 (0)	5 (6) 16 (20) 12 (15) 6 (8) 3 (4)	0.291 0.616 0.121 0.963 0.462
NY-ESO-1 IHC +ve IHC -ve	11 (79) 3 (21)	15 (19) 65 (81)	< 0.0001
Overall survival, mean (range) months All patients Adjuvant chemotherapy No chemotherapy	14.8 (0.6 – 37.5) 22.2 (9.4 – 37.5) 9.3 (0.6 – 35)	29.1 (0.03 – 146.5) 28.5 (0.60 – 117.1) 29.6 (0.03 – 146.5)	0.003 0.149 0.004

**Figure 4:**
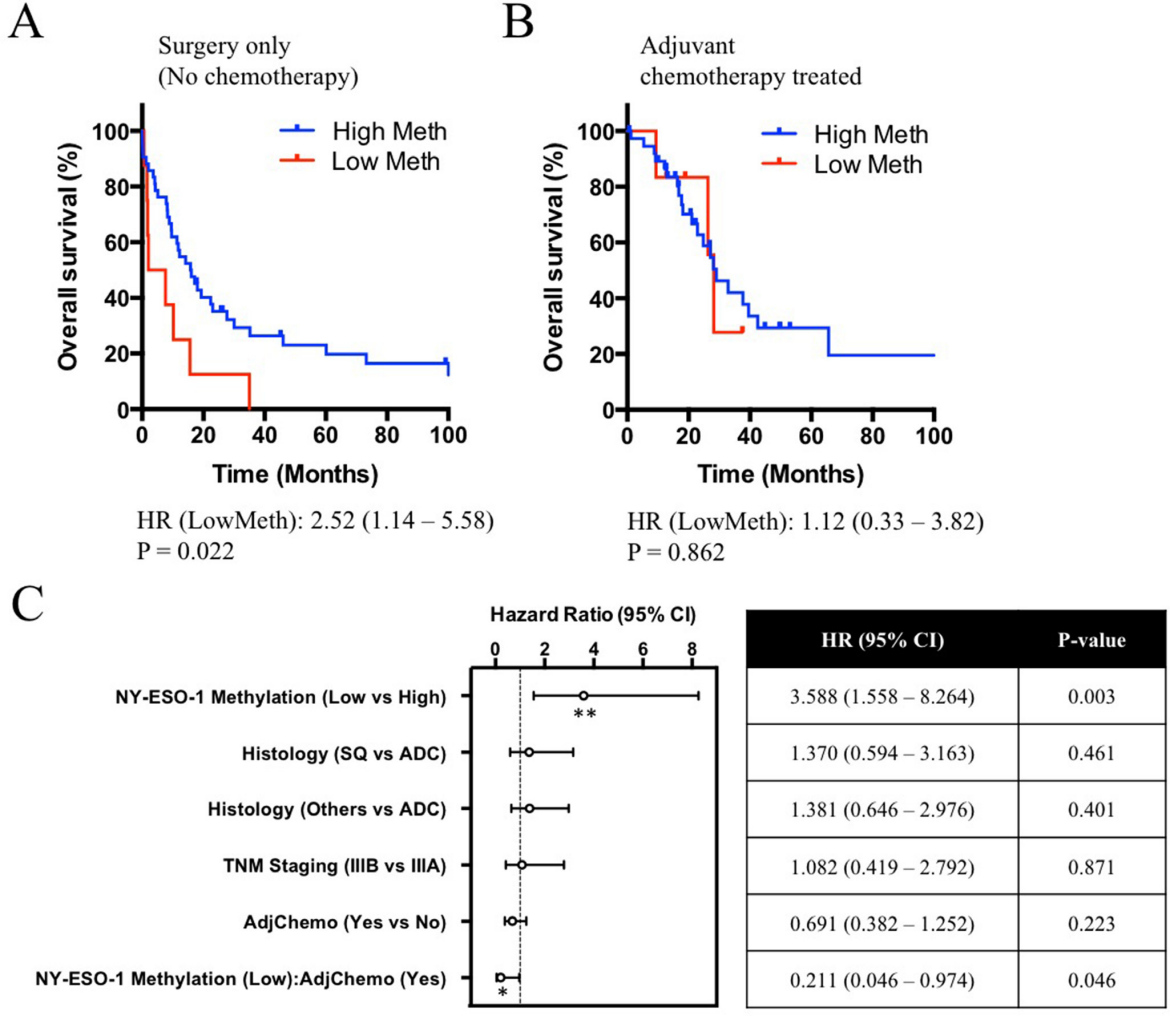
Clinical correlations of NY-ESO-1 promoter methylation and overall survival in patients underwent surgery alone or surgery and adjuvant chemotherapy Survival curves of patients with high versus low NY-ESO-1 methylation were compared using the log-rank Mantel-Cox test. (**A**) A significant trend (*P* = 0.022) was observed in the patients not receiving adjuvant chemotherapy, where patients with NY-ESO-1 low methylation showed worse overall survival. (**B**) However, in the patient group that was treated with chemotherapy, no difference in overall survival was observed between patients with high or low NY- ESO-1 promoter methylation. (**C**) Forest plot and a summary table detailing the clinicopathological features that are associated with overall survival of the patient cohort in a multivariate analysis. SQ, Squamous Cell carcinoma; ADC, Adenocarcinoma; AdjChemo, Adjuvant Chemotherapy.

To determine whether NY-ESO-1 promoter hypermethylation is prognostic and/or predictive of lung cancer patient survival or response to chemotherapy, univariate Cox regression analysis was performed and demonstrated a significant association with poorer survival (HR 2.52, 95% CI: 1.14 – 5.58, *P* = 0.022) in tumours with low NY-ESO-1 methylation when no adjuvant chemotherapy was used (Figure 4A and Supplementary Table 4). In the patient group that received adjuvant chemotherapy, no significant difference in overall survival was observed (Figure 4B and Supplementary Table 4). These results were similar to the outcome of a univariate Cox regression analysis performed to test the prognostic value of NY-ESO-1 protein expression as a marker for survival (HR 2.16, 95% CI: 1.13 – 4.09, *P* = 0.019) (Supplementary Table 5) [15]. In multivariate Cox regression analysis adjusting for stage, histology and chemotherapy NY-ESO-1 hypomethylation was an independent poor prognostic marker (HR 3.59, 95% CI: 1.56 – 8.26, *P* = 0.003), whereas treatment with chemotherapy and NY-ESO-1 hypomethylation was an independent predictor of improved survival (HR 0.211, 95% CI: 0.046 – 0.973, *P* = 0.046) (Figure 4C, Supplementary Table 6). Despite the poorer prognosis of patients with hypomethylated NY-ESO-1 promoter, these findings suggest that this patient subset may in fact respond better to cisplatin-based chemotherapy. Collectively, these results highlighted the potential use of NY-ESO-1 promoter methylation as both a prognostic biomarker of outcome, as well as a predictive biomarker of chemoresponse.

### Effect of NY-ESO-1 on chemosensitivity to cisplatin

To further investigate the link between NY-ESO-1 methylation status and cisplatin response, we performed MTS assays to determine the GI_50_ for cisplatin in the 14 lung cancer cell line panel. The GI_50_ for cisplatin was significantly lower (*P* = 0.043) in NY-ESO-1^+ve^ lines (40.1 ± 7.6 µM) compared to NY-ESO-1^-ve^ cell lines (102.9 ± 14.6 µM) (Supplementary Figure 9A, 9B). Consistent with this, a significant positive correlation between NY-ESO-1 expression and cisplatin sensitivity was observed (Supplementary Figure 9C, 9D).

Given the increased sensitivity to cisplatin in NY-ESO-1 positive cell lines, we investigated whether 5-Aza-dC-induced re-expression of NY-ESO-1 could enhance sensitivity to cisplatin in NY-ESO-1^-ve^, hypermethylated cell lines. Two cisplatin resistant hypermethylated cell lines A549 and NCI-H460 were used. NY-ESO-1 hypomethylation and re-expression at both mRNA and protein level was robustly induced by 5-Aza-dC treatment. However, there was no significant change in sensitivity to cisplatin following 5-Aza-dC treatment in both A549 and NCI-H460 (Supplementary Figures 4, 10A, 10B). To confirm these findings, we also performed siRNA-mediated knockdown of NY-ESO-1 in the NY-ESO-1^+ve^ SK-LC-17 and SK-LC-19 cells, followed by MTS assays. Transient transfection with NY-ESO-1 targeting siRNAs resulted in > 80% knockdown of NY-ESO-1 expression in both cell lines (Supplementary Figure 10C, 10D). However, no significant change in cisplatin-induced growth inhibition was observed after NY-ESO-1 knockdown (Supplementary Figure 10E, 10F). These results suggest that NY-ESO-1 does not play a functional role in mediating *in vitro* cisplatin-related chemo-sensitivity in lung cancer cell lines.

## DISCUSSION

We have demonstrated that NY-ESO-1 hypomethylation frequently occurs in lung cancer cell lines and tissue specimens and the relative level of methylation significantly correlated with both NY-ESO-1 mRNA and protein expression. NY-ESO-1 hypomethylation was an independent adverse prognostic marker in patients with NSCLC and demethylation of the NY-ESO-1 promoter was associated with re-expression of NY-ESO-1. Despite the association of NY-ESO-1 hypermethylation and consequential protein expression with chemosensitivity in patient samples, chemosensitivity of lung cancer cell lines to cisplatin treatment was unaffected by either 5-Aza-dC-induced promoter demethylation (and associated re-expression of NY-ESO-1) or siRNA-mediated knockdown of NY-ESO-1 *in vitro.* These results indicate that unknown extrinsic factors are present in patients (but absent in cell lines) that mediate chemosensitivity to cisplatin. One such factor could be the innate immune system and the associated cellular components. Alternatively, NY-ESO-1 is a non-functional surrogate marker for chemoresponse. Lastly, we did not demonstrate an association between PD-L1 expression and NY-ESO-1 methylation however of considerable interest was the finding that 5-Aza-dC induces not just NY-ESO-1 expression but also PD-L1 expression in a significant number of NSCLC cell lines.

These data are consistent with previous reports [8, 18], demonstrating that NY-ESO-1 hypomethylation and resulting protein expression is a poor prognostic marker in NSCLC. The use of quantitative MS-PCR technology enables the robust detection of gene-specific methylation with the need for only small quantities of tissues from patients. Comparing to the traditional standard-of-care molecular pathology tools such as the IHC, the MS-PCR approach enable a more precise quantitative measurements for comprehensive statistical analysis (i.e. the regression analysis to determine the predictive power of potential biomarkers). Indeed, in our current study, we have demonstrated that the NY-ESO-1 methylation status can be used to accurately predict NY-ESO-1 expression in patient specimens. Clearly, when tissue samples are limited, this approach may represent a very useful assay for candidate gene markers that are regulated by promoter methylation at the transcriptional level.

Despite not reaching statistical significance, there is a larger number of adenocarcinoma (ADC) exhibiting NY-ESO-1 promoter hypermethylation when compared to that of squamous cell carcinoma (SCC). Given promoter hypermethylation leads to epigenetic silencing, it is therefore predicted that a larger proportion of SCC would be NY-ESO-1 positive when compared to ADC. In fact, this prediction held true based on the findings made in our prior study [15] and is in close agreement to what was previously reported in several independent lung cancer patient cohorts [8, 9, 18–20]. Interestingly, SCC is not only more likely to express NY-ESO-1 but also appears to respond better to immunotherapy, regardless of PD-L1 expression [21, 22].

While the negative association of CTA expression and patient survival has been consistently reported [8, 15, 18], the biological explanation for these observations remains elusive. However, the co-expression of these CTAs hinted at the possibility that these phenomena were part of a global demethylation event, which leads to not only aberrant expression of CTAs but also other oncogenes. Clearly, CTA expression is associated with more advanced disease stages, perhaps reflecting more widespread epigenetic alterations as cancer progresses. The immunogenicity of these proteins may have functional relevance given the recent excitement with immune checkpoint inhibitors [11]. Although we did not find a significant association between NY-ESO-1 and PD-L1 expression, the induced expression of PD-L1 in negative cell lines by 5-Aza-dC treatment, suggests that global demethylation may trigger aberrant expression of immunological markers such as PD-L1 *in vitro*.

Utilizing NY-ESO-1 or demethylating agents as a combination strategy with checkpoint inhibition in NSCLC, may still hold promise. NY-ESO-1 is one of the most immunogenic CTAs, making it an attractive target for cancer vaccines [10]. It has been used as a tumour vaccine in a variety of tumour types, in particular in melanoma [23–25] with effective T-cell responses post vaccination, albeit without significant clinical improvement in the disease. Combination approaches may offer another mechanisms, by which the immunoregulatory brakes are removed while also activating tumour specific T-cells. Certainly, the clinical effect of T-cell activation has been effectively demonstrated using adoptive T-cells stimulated towards NY-ESO-1 epitopes [26].

The fact that *de novo* but not induced NY-ESO-1 expression modulates chemosensitivity is worthy of further investigation. We had previously postulated that the survival differences associated with patients undergoing chemotherapy who were NY-ESO-1 positive may have been mediated by an immune response to treatment [15]. Certainly, the immunogenicity of CTAs has previously been demonstrated and that the effect of tumor lysis as an immune primer is feasible. However, our results indicate that lung cancer cell lines expressing NY-ESO-1 are more sensitive to chemotherapy, even in the absence of an intact immune system. Additionally, our *in vitro* data suggests that NY-ESO-1 itself does not play a direct functional role in determining chemosensitivity, as both 5-Aza-dC-induced re-expression or RNAi-mediated knockdown of NY-ESO-1 failed to alter chemosensitivity. However, it should be noted that the sample size used in determining the association of *in vitro* chemosensitivity and NY-ESO-1 protein expression is small. Therefore, experimental results from a larger panel of lung cancer cell lines are required before a solid conclusion could be made in this regard.

There are several potential explanations for our findings. First, tumours that express NY-ESO-1 have undergone extensive epigenetic reprogramming resulting in the coordinated re-expression of many other genes that alter chemosensitivity [27]. However, the level of NY-ESO-1 re-expression *in vitro* appears to be significantly lower when compared to cell lines with *de novo* NY-ESO-1 expression. 5-Aza-dC is cytotoxic agent *in vitro* and it is possible that our assay failed to detect robust changes in chemosensitivity due to inadequate NY-ESO-1 re-expression. In part, this could be improved with multiple, continual low dose exposure to 5-Aza-dC or combining 5-Aza-dC with a clinical histone deacetylase inhibitor such as Vorinostat to further enhance NY-ESO-1 expression to investigate its role in regulating chemosensitivity.

Our cell line data demonstrated robust NY-ESO-1 promoter demethylation and consequential induction of NY-ESO-1 mRNA expression when tumour cells were treated with 25–100 nM 5-Aza-dC for a continuous period of 3 days. Unlike other previous studies [9, 28, 29], we have also demonstrated re-expression of NY-ESO-1 at the protein level. However, with the exception of NCI-H520 which demonstrated re-expression in > 80% of total cell population, the re-expression was restricted to < 20–30% of the total cell population in majority of the tumour cell lines analyzed (e.g. NCI-H460, A549, NCI-H838, NCI-H522 and SK-LC-05). It is currently unknown as to why 5-Aza-dC-mediated NY-ESO-1 re-expression at protein level was not observed in majority of the total cell population. However, one possible explanation is that the observation is likely to reflect a pattern of heterogenous promoter methylation of NY-ESO-1 in a given cell population. As a result, the concentration of 5-Aza-dC and the duration of treatment used was only sufficient to induce NY-ESO-1 demethylation and protein re-expression in cells exhibiting low level of NY-ESO-1 methylation. By comparison, complete demethylation and protein re-expression in cells exhibiting high levels of NY-ESO-1 methylation is expected to come from treatment using 5-Aza-dC at higher doses, with longer duration, or in combination with HDAC inhibitors (that acetylate histones at the promoters).

Recently, Odunsi and colleagues presented a novel immune-chemotherapy approach showing that 5-Aza-dC increased the effectiveness of an NY-ESO-1 vaccine in 12 patients with platinum refractory ovarian cancer [30]. Moreover, a small study in NSCLC suggested that epigenetic therapy may sensitize cancers to immune checkpoint therapy targeting PD-L1/PD-1 interaction [31]. Six patients who received treatment on a clinical trial of epigenetic therapy were placed on trials for anti PD-1 and PD-L1 immunotherapy with three achieving durable partial and two stable disease. At the cellular level, the mechanism by which epigenetic priming of immunotherapy induces improved outcomes may involve upregulation of gene clusters associated with interferon-signaling, antigen processing, as well as genes encoding CTAs [14, 31]. Our findings also suggest that PD-L1^-ve^ tumors may become positive through the use of demethylating agents. Indeed, 5-Aza-dC treatment has previously shown to induce PD-L1 demethylation and consequential mRNA and/or protein re-expression in the KG1 leukemia cell line, *in vitro* activated primary CD8+ T cells, CD4+ and CD8+ T cells from patients with myelodysplastic syndrome and acute myeloid leukemia [32–34]. Given 5-Aza-dC-mediated PD-L1 demethylation has been reported in these previous studies and that 5-Aza-dC is a well-established demethylating agent, it is therefore unlikely that 5-Aza-dC-mediated PD-L1 re-expression occurs independent of PD-L1 promoter demethylation. Importantly, the hypothesis of combining epigenetic therapy together with immune checkpoint inhibitors may shift the balance toward enhanced adaptive and innate immune response and is currently being investigated in a prospective phase II clinical trial (NCT01928576).

High mutational load and neoantigen expression has been shown to predict for responses to immune checkpoint inhibition [35]. In this context, it is plausible that global demethylation, which frequently occurs in malignancies, may result in (re)expression of genes or neo-antigens that were previously silenced. This could explain the superior responses observed in patients treated with PD-1 inhibitors following administration of demethylating agents [31, 36]. Clearly, the concept of tumor lysis exposing immunogenic proteins and/or neo-antigens remains plausible when considering these early data.

The field of immunotherapy is rapidly changing and predictors of therapeutic benefit are increasingly important. The use of demethylating agents in some diseases has shown benefit, but in most solid organ cancers it has been largely speculative. It remains to be seen whether targeting tumours with a *de novo* unmethylated phenotype with immunological agents may improve outcomes. However, our data suggest that combination chemo-immuno therapies may provide an important strategy for these tumours, especially given their poorer clinical outcomes.

## MATERIALS AND METHODS

### Patients and specimens

A total of 99 tumour samples were obtained from two tertiary hospitals (Austin and St Vincent’s Hospitals, Melbourne, Australia) for patients who had been treated for pathological N2 (pN2)/stage 3 NSCLC. Medical records were retrospectively reviewed for clinicopathological characteristics. The resected primary specimens and associated nodal tissues were used. Patients were staged pre-operatively with positron emission tomography (PET) scans or mediastinoscopy. Some of these patients were diagnosed prior to the broad acceptance of adjuvant chemotherapy resulting in two cohorts: 45 (45.5%) patients received four cycles of platinum-based doublet chemotherapy and 54 (54.5%) did not. The study was approved by the Austin Hospital’s Human Research Ethics Committee.

### Cell culture

A panel of 14 lung cancer cell lines were used: NCI-H2170, A549, NCI-H522, HCC 827, NCI-H460, NCI-H520, NCI-H1975, and NCI-H1650, were purchased from American Type Culture Collection (ATCC), while SK-LC-02, SK-LC-05, SL-LC-15, SK-LC-17, and SK-LC-19, were provided by Prof. Gerd Ritter at the Memorial Sloan-Kettering, and NCI-H838 by Prof. Neil Watkins from Monash Institute of Medical Research, Australia (Supplementary Table 1). All cell lines have been tested and authenticated using GenePrint®10 System (Promega, USA). Cells were grown in a recommended medium culture in a humidified incubator with 5% CO_2_ at 37°C.

### Immunohistochemistry

Protein expression of NY-ESO-1 was determined by immunohistochemistry (IHC) analysis on formalin-fixed paraffin embedded (FFPE) tissues [15] and tissue culture tumour cell lines [37] as previously described. Briefly, 4-µm sections were cut and stained with the murine monoclonal anti-NY-ESO-1 antibody (clone E978) obtained from the Ludwig Institute for Cancer Research and used at a final concentration of 2.5 µg/mL. PD-L1 positivity was defined as > 5% cells with membranous staining of intensity and strong positivity was defined as > 50% cells with membranous staining of intensity. Testicular tissue sections were used as positive controls. Normal lung tissues and absence of primary antibodies were used as negative controls. PD-L1 staining was performed using a rabbit monoclonal anti-PD-L1 antibody (E11340 XP, Cell Signaling Technology), as previously described [38].

### Bisulphite treatment

Genomic DNA (gDNA) was isolated from lung cancer cell lines and archival FFPE tissues using DNeasy Kits (Qiagen), in accordance with the manufacturer’s instructions. Bisulfite conversion of the DNA samples was performed as previously described [39]. The quantity and quality of the gDNA was measured using a NanoDrop 2000 Spectrophotometer (Thermo Scientific). For bisulfite treatment, 1 µg of isolated gDNA was converted using the EpiTect Bisulfite Kit (Qiagen), according to the manufacturer’s instructions. For subsequent methylation analysis, DNA was eluted with RNAase-free water to a final concentration of 25 ng/μL.

### Quantitative methylation specific-PCR

Primers utilized for quantitative methylation specific-PCR (qMS-PCR) were previously described and validated [17]. The primer sequences are provided in the Supplementary Table 2. The locations of primers relative to the CpG island of the NY-ESO-1 (encoded by the CTAG1B gene) promoter were determined by webgene (http://www.itb.cnr.it/sun/webgene/) using published criteria (Supplementary Figure 1A) [40]. Primers targeting the converted DNA sequences of beta-actin (ACTB) gene were used as normalization controls in MS-PCR reactions [41]. PCR reactions were performed as previously described [42, 43] and in duplicate in a final volume of 10 μl using 50 ng bisulfite-converted DNA with 10 mM forward and reverse primers, and 1 μl RNAase-free water. 7500 FAST real time cycler (Applied Biosystems) was used with the following cycling conditions: 1 cycle of 95 °C for 10 minutes, 50 cycles of 95°C for 15 seconds and 60 °C for 1 min.

To determine the relative level of NY-ESO-1 methylation present in each sample, two different methods were used: (1) expressed as the percentage of methylation (%M); (2) reported as the ratio of methylated (M) to unmethylated (UM) molecules for a particular locus, as a Log_2_ (M/UM) ratio [44]. Both methods were based on the use of threshold cycle (C_T_) of the NY-ESO-1 gene and of the internal reference gene, beta-actin (ACTB). To determine the relative amount of methylated or unmethylated alleles in each sample, 2^-dCT^ method was employed, where dC_T_ (M) = [C_T_ (NY-ESO-1 methylated) - C_T_ (ACTB)]; and dC_T_ (UM) = [C_T_ (NY-ESO-1 unmethylated) - C_T_ (ACTB)]. Methylation percentage in the samples were calculated by M/(M+UM) x 100, where M is the quantity of methylated NY-ESO-1 alleles, and UM is the quantity of unmethylated NY-ESO-1 alleles, respectively. In comparison, Log2 (M/UM) is calculated by Log2 [2^-dCT^ (M) / 2^^-dCT^ (UM)].

The patient sample population was separated into NY-ESO-1 methylation low or high groups. An optimal functional cut-off of Log2 fold-difference in M/UM of ≥ 5 was determined by ROC curve analysis (Supplementary Figure 2). Samples that has a Log2 (M/UM) ≥ 5 is classified into the NY-ESO-1 ‘High Meth’ group, while a Log2 (M/UM) < 5 was classified into NY-ESO-1 ‘Low Meth’ group. Importantly, 94% (64/68) of NY-ESO-1 negative tumours were classified into the group containing low methylation (i.e. hypomethylation), demonstrating the predictive power and the robustness of the cut-off used. Using this selection criterion, 84% of tumour specimens (79/94) were classified as NY-ESO-1 ‘methylation high’ (i.e. hypermethylated) and 16% (15/94) were classified as NY-ESO-1 ‘methylation low’ (i.e. hypomethylated).

### Quantitative RT-PCR

Quantitative RT-PCR (qRT-PCR) was performed as previously described [45, 46]. Total RNA was extracted from exponentially growing cells using RNeasy Mini Kit (Qiagen) according to the manufacturer’s instructions. cDNA synthesis was performed using Transcriptor First Strand cDNA Synthesis Kit with random hexamer (Roche). qRT-PCR was carried out using Power SYBR Green PCR Master Mix (Life Technologies) on 7900HT Fast Real-Time PCR System (Life Technologies) according to the manufacturer’s instructions. cDNA equivalent to 10 ng RNA was amplified with 75 nM forward and 75 nM reverse primers in 10 µL reaction. Dissociation curves were performed to confirm specific amplifications without primer dimer formation. Samples were also subjected to gel electrophoresis analysis to confirm that the PCR products were of expected size. Abundance of mRNA expression was determined using the comparative C_T_ method and expressed relative to ACTB expression.

### RNAi-mediated knockdown

Transient transfection of lung adenocarcinoma cell lines, SK-LC-17 and SK-LC-19, with 10 nM of non-targeting control siRNA and specific siRNA duplexes targeting NY-ESO-1 (s194368, Ambion by Life Technology) was performed using Lipofectamine RNAiMAX in 24 well plate format, according to the manufacturer’s instructions (Invitrogen, USA). Twenty-four hours post transfection, cells were collected to test for knockdown efficiency and re-seeded into 96 well plates for MTS assays.

### Treatment with 5-Aza-2′-deoxycytidine (5-Aza-dC)

∼1x10^6^ cells were seeded in T-25 flask and treated with 5-Aza-2’-deoxycytidine (Sigma) for 3 consecutive days at a concentration of 0.1 µM. Cells were washed with 1X PBS and incubated with fresh medium for a 72-hour recovery period, and subsequently transferred into 96-well plates for MTS assays. In parallel, cells were harvested for RNA and DNA isolation for qRT-PCR and qMS-PCR, respectively.

### MTS assays

MTS assays were performed as previously described [47] using CellTiter 96 (Promega, Madison, USA). Post 5-Aza-dC treated or NY-ESO-1 knockdown lung cancer cell lines were independently seeded in 96-well plates (7500 cells per well) and treated with 0.1 µM to 1 mM cisplatin for 72 hours. Changes in relative proliferation were estimated by measuring the absorbance at 490 nm in a microplate reader.

### Statistical analysis

Fisher’s exact test was used to evaluate the association between NY-ESO-1 promoter methylation and NY-ESO-1 protein expression. Univariate and multivariate Cox proportional hazard ratio modeling analyses were performed in R (v3.0.1, survival package v2.37–4) and STATA (version 12). Student’s two-tailed *t*-test was performed in Prism 6 (GraphPad Software Inc).

## SUPPLEMENTARY MATERIALS TABLES AND FIGURES



## Abbreviations

5-Aza-dC: 5-aza-2′-deoxycytidine
ACTB: actin beta
CT: cancer/testis
CTA: cancer/testis antigens
CTAG1B: cancer/testis antigen 1B
MS-PCR: methylation specific-polymerase chain reaction
NSCLC: non-small cell lung cancer
NY-ESO-1: New York esophageal squamous cell carcinoma-1
PET: positron emission tomography
PD-L1: programmed death-ligand 1
