# Supplementary material for: Promoter hypomethylation of NY-ESO-1, association with clinicopathological features and PD-L1 expression in non-small cell lung cancer
Source: Oncotarget. 2017 May 23;8(43):74036–48. doi: 10.18632/oncotarget.18198 (PMC5650321; doi:10.18632/oncotarget.18198)
Supplement: Supplementary file 1 [file oncotarget-08-74036-s001.pdf]

Promoter hypomethylation of NY-ESO-1, association with clinicopathological features and PD-L1 expression in non-small cell lung cancer

SUPPLEMENTARY MATERIALS

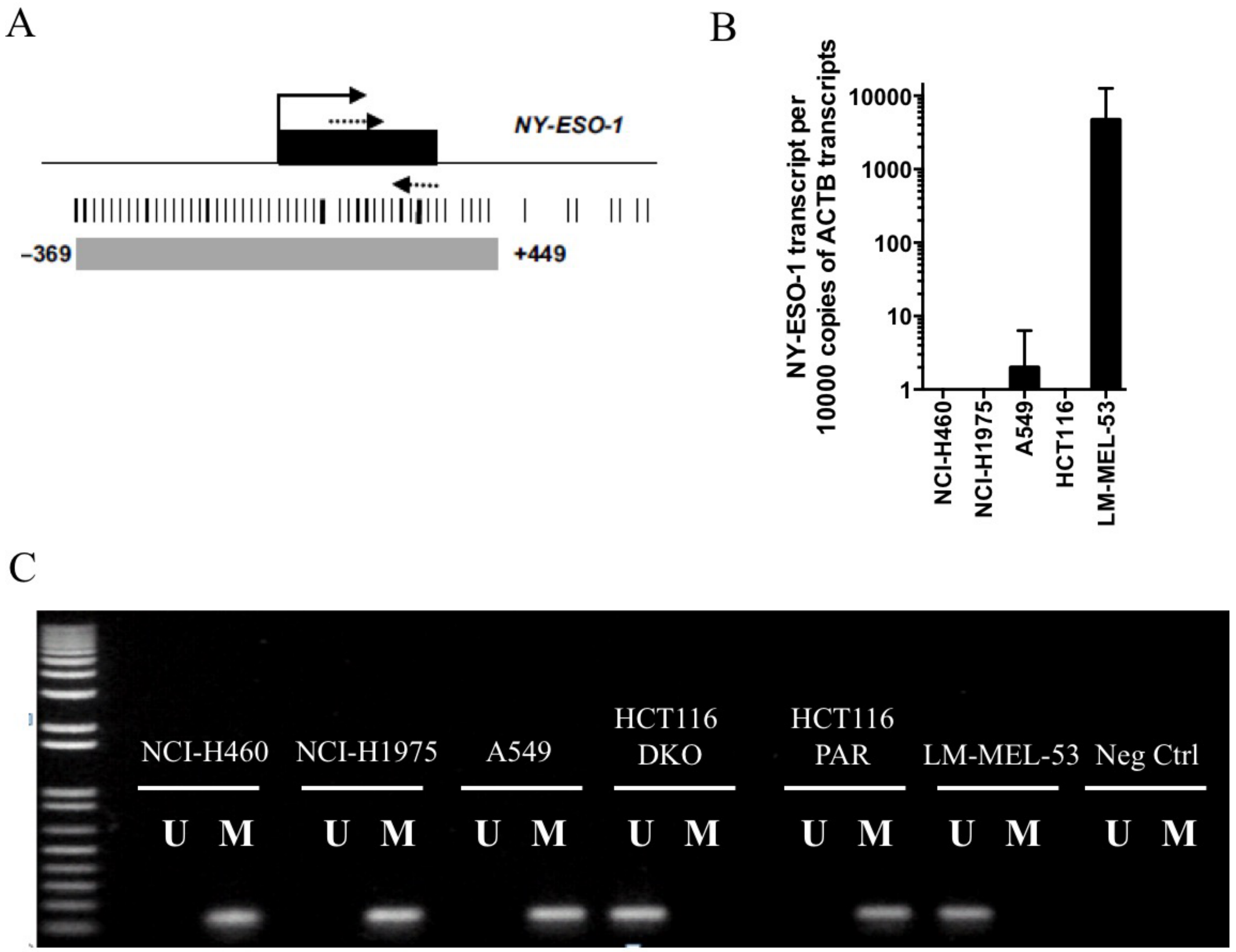

Supplementary Figure 1: Quantitative MS-PCR and RT-PCR analysis. (**A**) 5' CpG island within NY-ESO-1 gene promoter. The CpG island of NY-ESO-1 was defined as described in Materials and Methods. NY-ESO-1 CpG island statistics: 818 bp, 71% GC, observed/expected CpG ratio = 0.58. Bent arrows indicate transcriptional start sites (TSS), black rectangles indicate the first exon (5′ UTR), gray rectangles indicate the 5' CpG, vertical lines indicate CpG dinucleotides, and broken arrows indicate the location of quantitative MS-PCR primers. Numbers indicate nucleotide positions relative to the TSS, indicated as position +1. Figure adapted from James et al., 2006 Oncogene. (**B**) NY-ESO-1 mRNA expression assessed by quantitative RT-PCR. (**C**) MS-PCR analysis in 3 NSCLC cell lines (NCI-H460, NCI-H1975 and A549) demonstrating hypermethylation of the NY-ESO-1 gene promoter, with a predominant PCR band amplified using oligonucleotide primers targeting the methylated (M) and not the unmethylated (UM) allele. Double knocked out (DKO) of DNMT1 and DNMT3B genes in HCT116 resulted in loss of NY-ESO-1 methylation as compared to the parental wild-type HCT116 colon carcinoma cell line, which is known not to express NY-ESO-1 due to promoter hypermethylaton. LM-MEL-53 expresses high levels of NY-ESO-1 and has a hypomethylated promoter, with predominant PCR band amplified using primers targeting the unmethylated (UM) allele. No DNA template control was included in the MS-PCR analysis as the negative control.

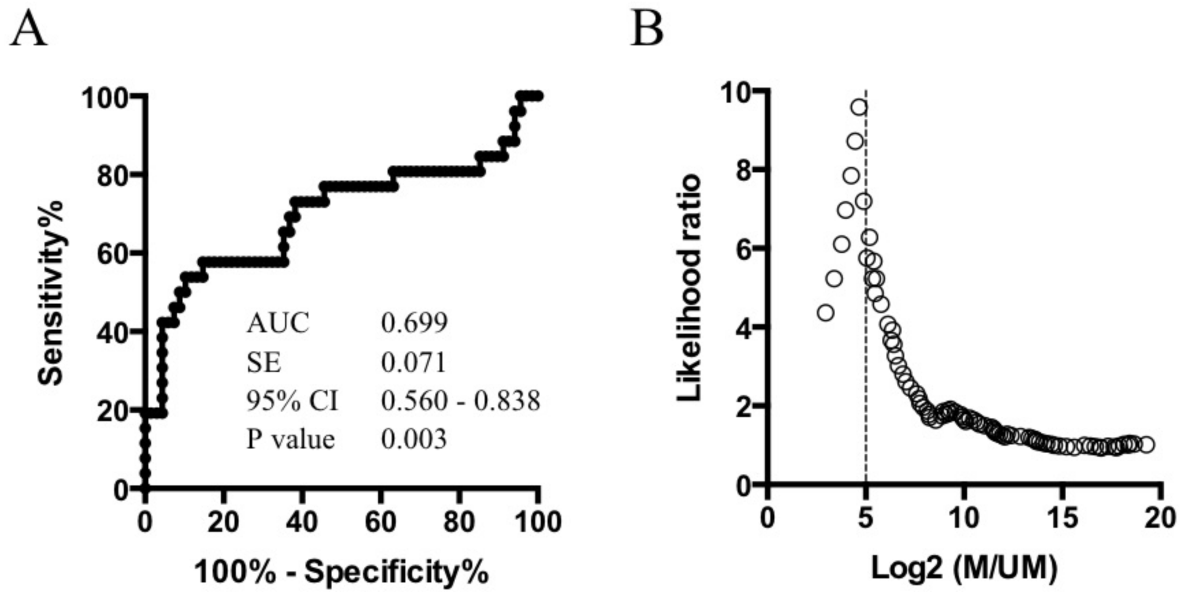

Supplementary Figure 2: Receiver operating characteristic (ROC) curve analysis. (**A**) ROC curve analysis was performed to determine the optimal functional cut-off of Log2 ratio (M/UM) of NY-ESO-1 in predicting NY-ESO-1 protein expression. (**B**) Likelihood ratios for positive results were plotted against the use of different cut-off of Log2 ratio (M/UM) of NY-ESO-1. An optimal functional cut-off of Log2 fold-difference in M/UM of ≥ 5 was determined and used to classify lung cancer patient samples into the NY-ESO-1 'High Meth' and 'Low Meth' groups. AUC, area under the curve; SE, standard error; CI, confidence interval.

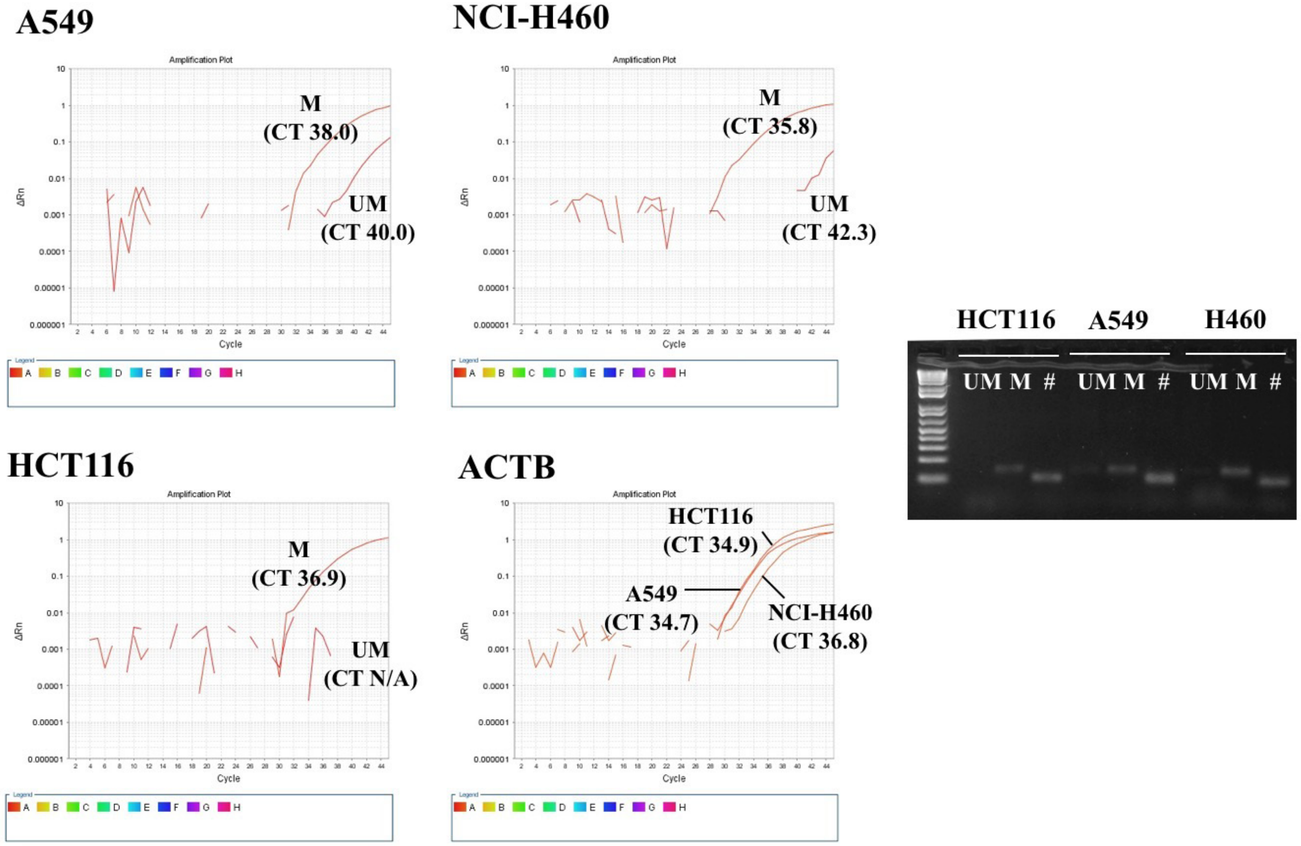

Supplementary Figure 3: Quantitative MS-PCR for NY-ESO-1 methylation detection. Representative amplification plots for the qMS-PCR analysis performed for three tumour cell lines A549, NCI-H460 and HCT116 were shown. CT (threshold cycle) for primers detecting methylated NY-ESO-1 allele (M), unmethylated NY-ESO-1 allele (UM), and control gene ACTB (beta-actin) was calculated and depicted in the amplification plots. Right panel includes a gel images showing specific amplified PCR products in qMS-PCR analysis. #, beta-actin control.

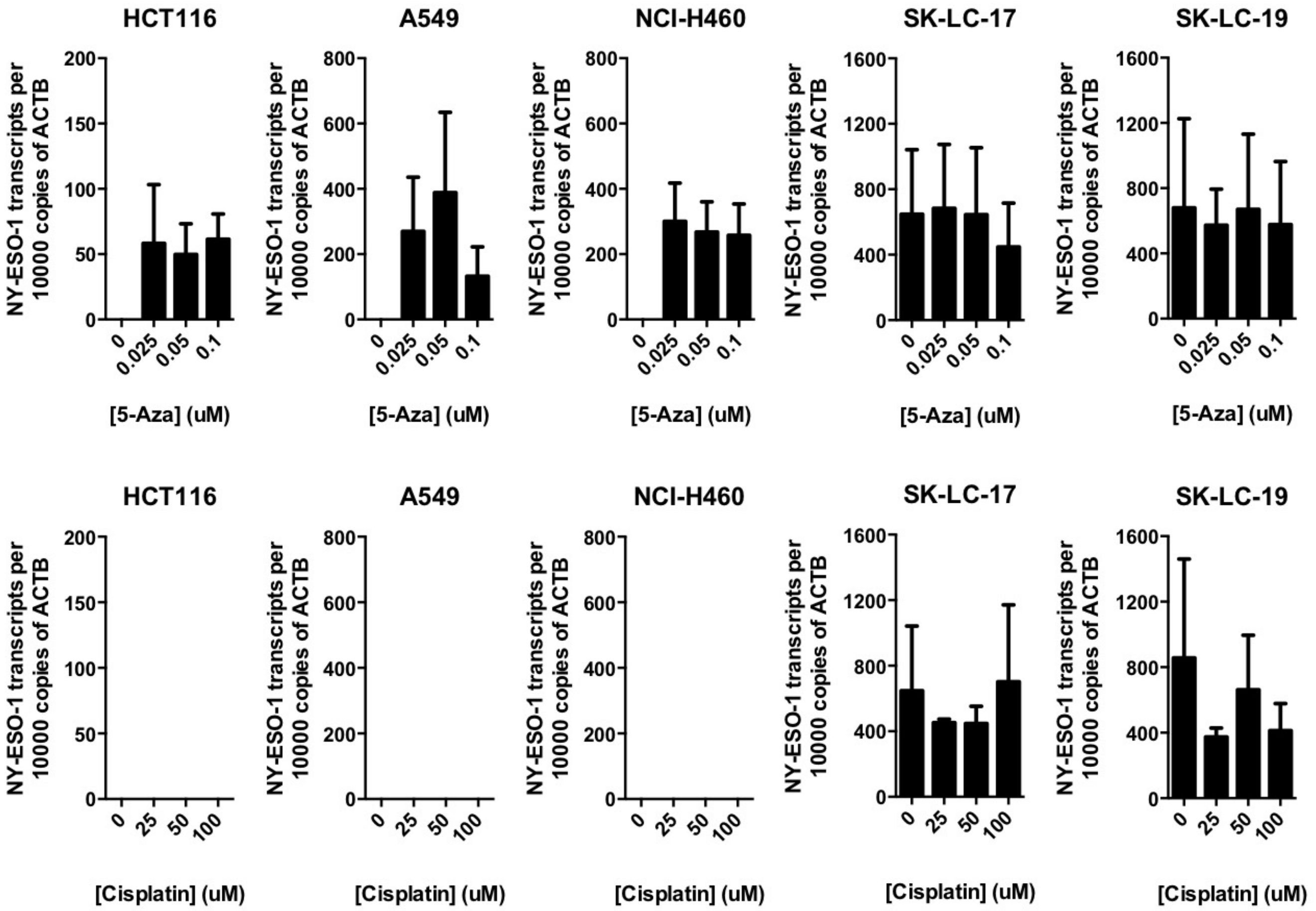

Supplementary Figure 4: 5-Aza-dC treatment on lung cancer cell lines resulting in NY-ESO-1 mRNA re-expression. Upper panel: Cell lines were treated with 0.025-0.1 µM 5-Aza-dC for 72 hr. 5-Aza-dC treatment resulted in re-expression of NY-ESO-1 at the mRNA level in hypermethylated, NY-ESO-1^{-ve} cell lines HCT116, A549, and NCI-H460. In contrast, no change in NY-ESO-1 mRNA expression was observed in hypomethylated, NY-ESO-1^{+ve} cell lines SK-LC-17 and SK-LC-19. Lower panel: Cisplatin treatment (25–100 µM, 72 hr) on the same panel of cell lines was performed, and no changes in NY-ESO-1 mRNA expression was observed.

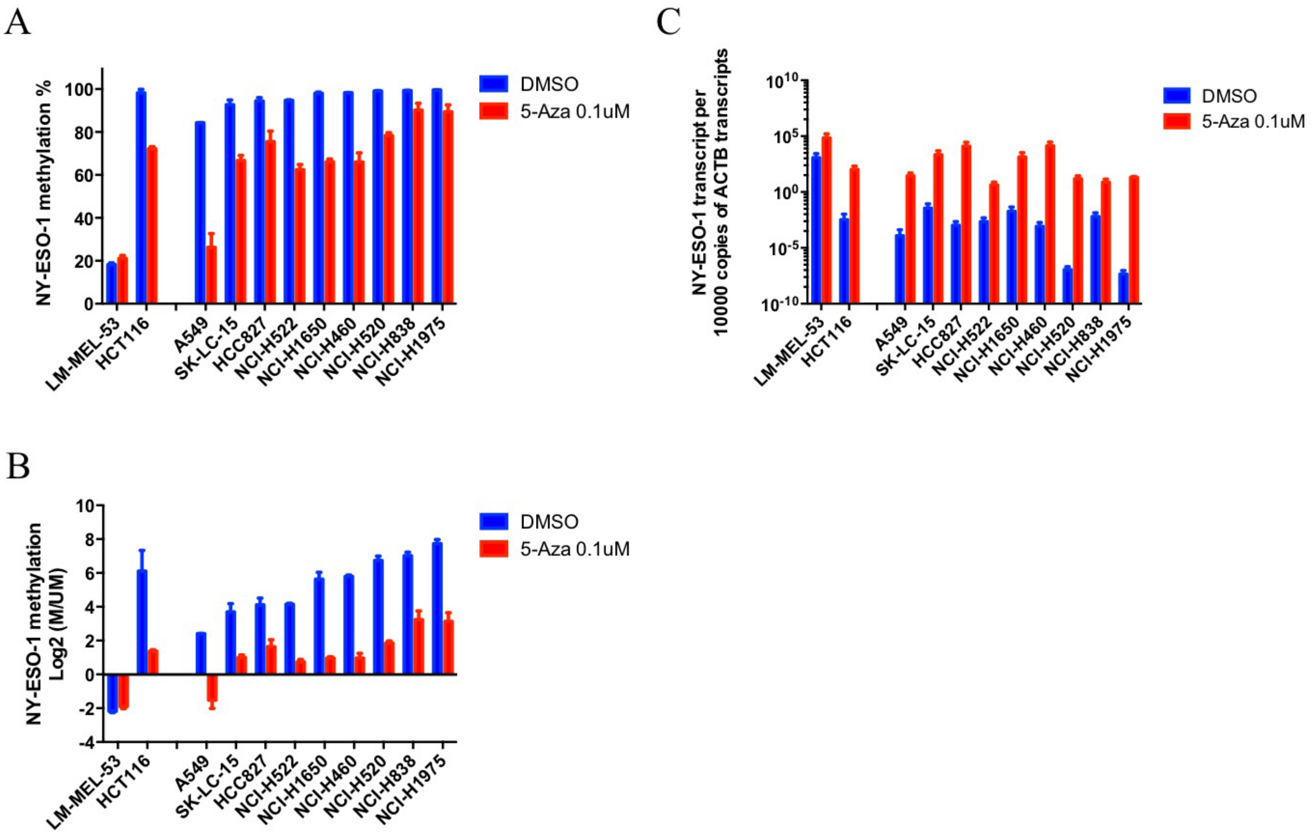

Supplementary Figure 5: 5-Aza-dC treatment on 9 NY-ESO-1 hypermethylated lung cancer cell lines resulting in NY-ESO-1 hypomethylation and mRNA re-expression. (**A–B**) Hypomethylation of NY-ESO-1 promoter following by 72 hr of 5-Aza-dC treatment at 0.1 μM. (**C**) mRNA expression of NY-ESO-1 in cancer cell lines treated with or without 0.1 μM 5-Aza-dC. Corresponding increase in NY-ESO-1 mRNA expression was observed in all 9 NY-ESO-1 hypermethylated lung cancer cell lines. 5-Aza-dC-mediated re-expression of NY-ESO-1 was also observed in NY-ESO-1 hypermethylated HCT116 cells but not hypomethylated LM-MEL-53 cells.

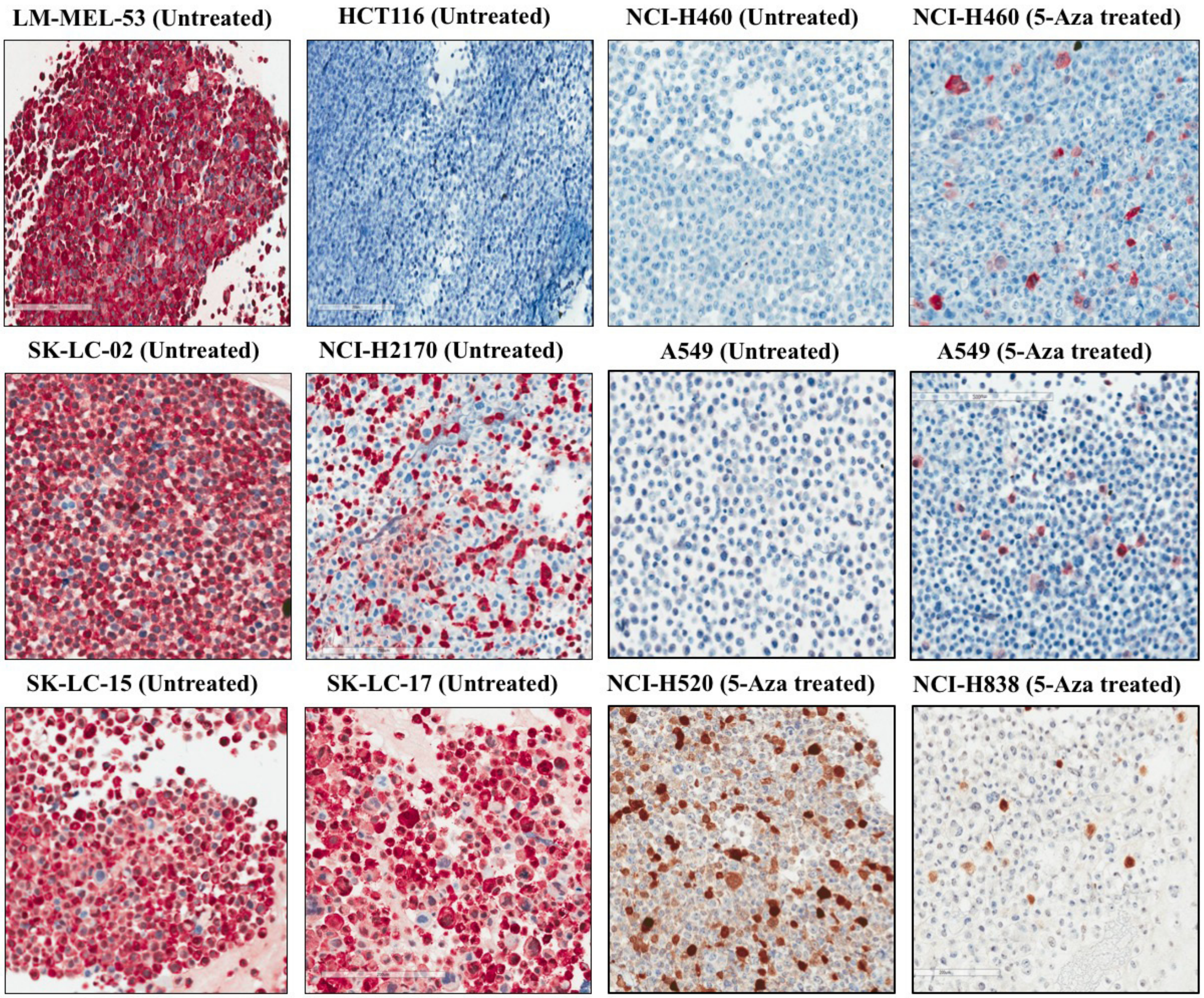

Supplementary Figure 6: Representative IHC staining for NY-ESO-1 using an anti-NY-ESO-1 (E978) antibody on melanoma (LM-MEL-53), colon carcinoma (HCT116) and lung cancer cell lines (SK-LC-02, SK-LC-15, NCI-H1270 and SK-LC-17). NY-ESO-1 negative cell lines, NCI-H460 and A549 were treated with 5-Aza-dC, resulting NY-ESO-1 upregulation post treatment. Re-expression of NY-ESO-1 in NY-ESO-1 negative cell lines, NCI-H520 and NCI-H838, was also observed post 5-Aza-dC treatment (untreated samples not shown).

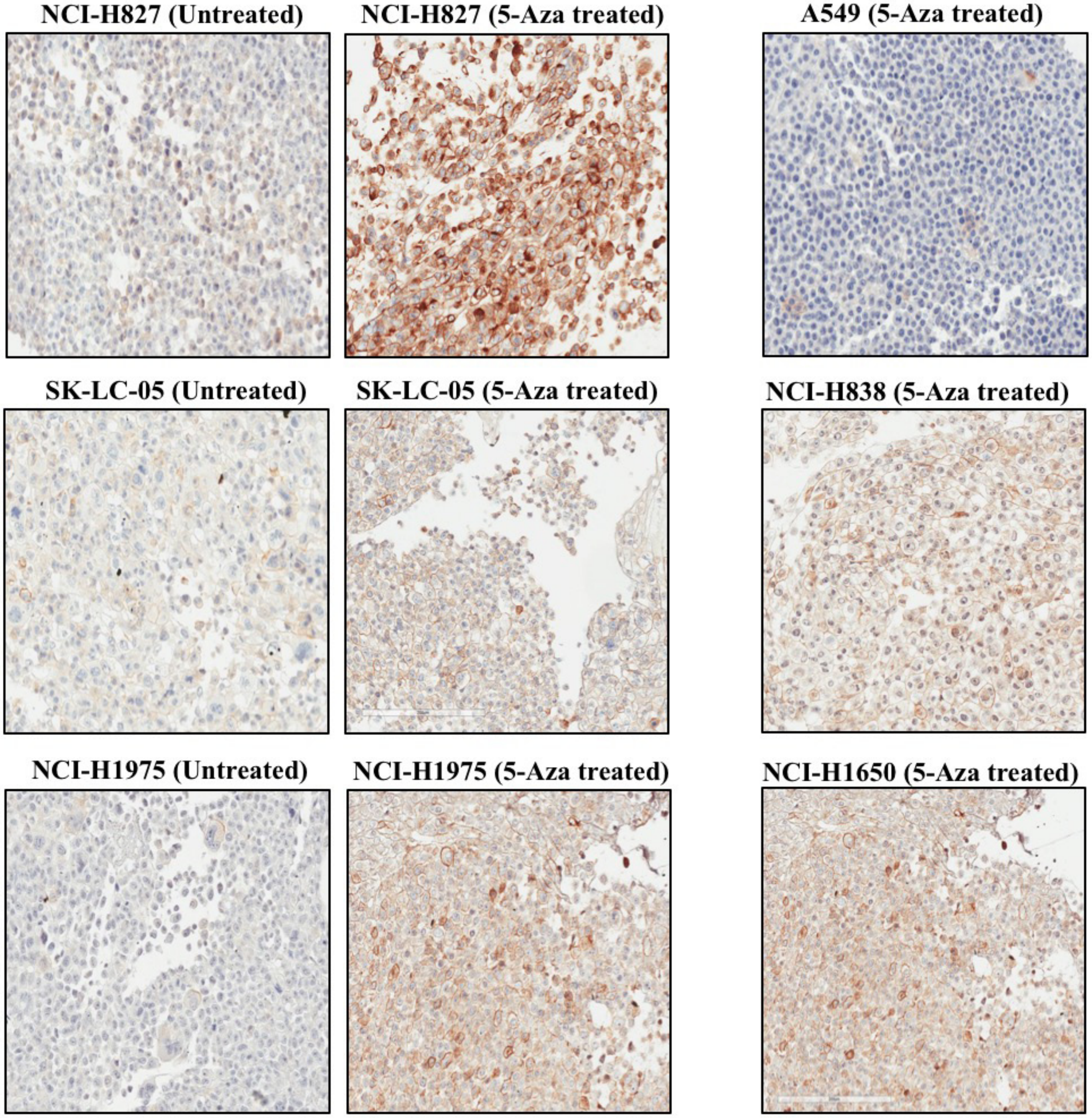

Supplementary Figure 7: Representative IHC staining for PD-L1 using an anti-PD-L1 (E11340 XP, Cell Signaling Technology) antibody on three lung cancer cell lines (NCI-H827, SK-LC-05 and NCI-H1975) pre and post 5-Aza-dC treatment. 5-Aza-dC treatment resulted in re-expression of PD-L1. PD-L1 re-expression was also observed in three additional lung cancer cell lines (A549, NCI-H838 and NCI-H1650) post 5-Aza-dC treatment (untreated specimens not shown).

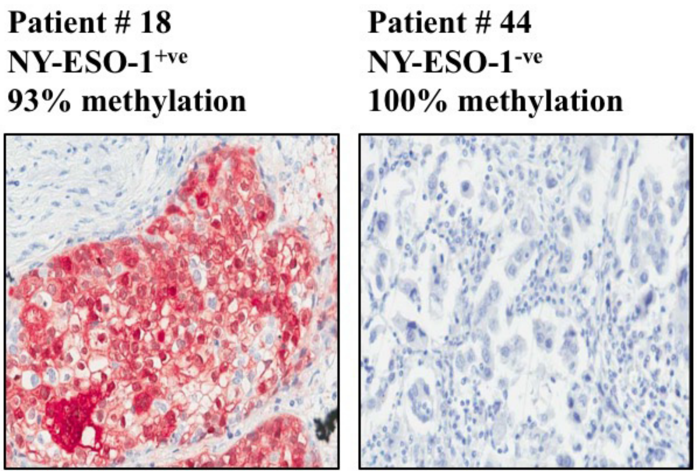

Supplementary Figure 8: Representative IHC staining for NY-ESO-1 with E978 antibody on tumour specimens derived from NY-ESO-1[+ve] patient #18 with 93% NY-ESO-1 methylation (left) and NY-ESO-1[-ve] patient #44 with 100% NY-ESO-1 methylation (right).

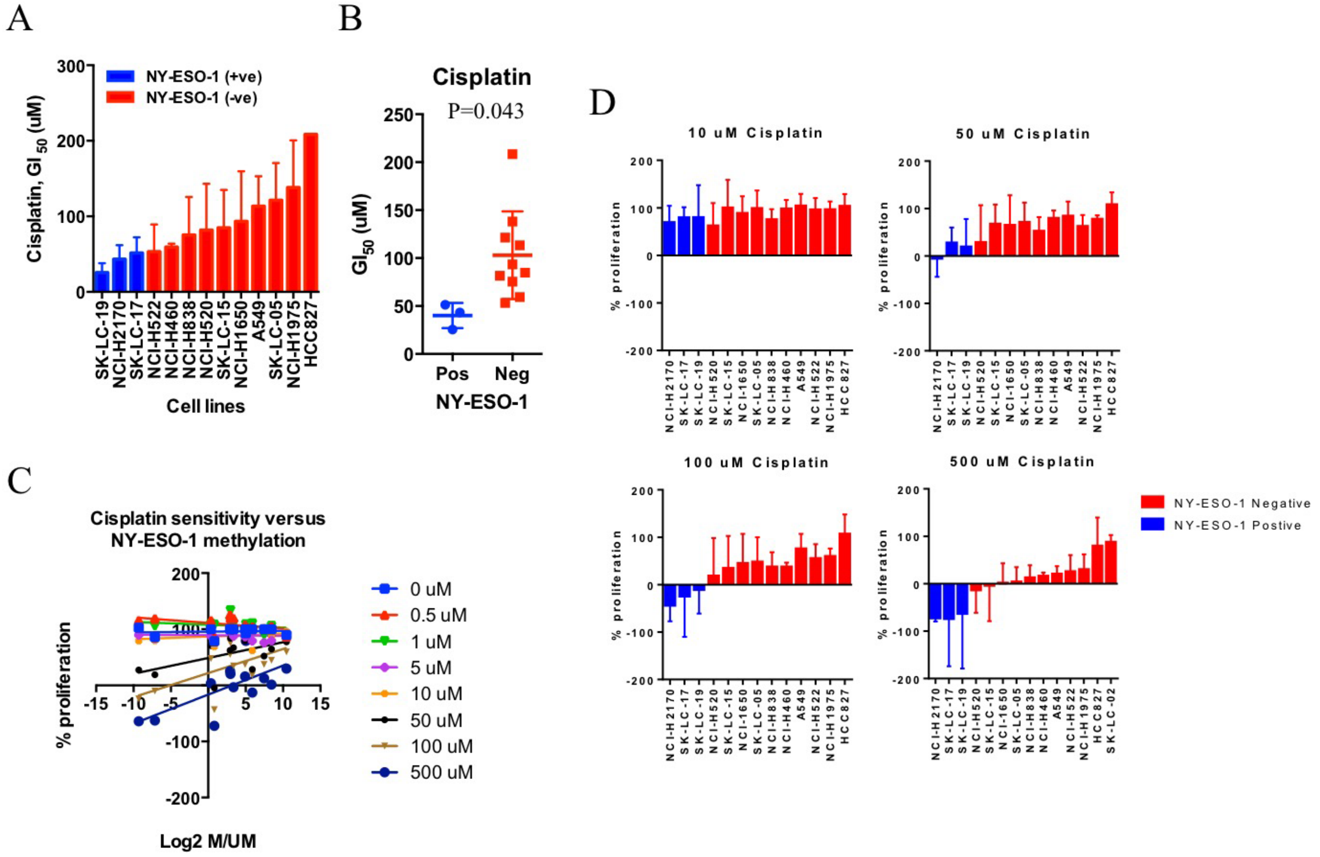

Supplementary Figure 9: Cisplatin sensitivity versus NY-ESO-1 methylation and expression in the lung cancer cell line panel. (**A–B**) Mean GI50 levels of 13 lung cancer cell lines. SK-LC-02 was not included in the analysis due to low proliferation growth. The mean GI50 is significantly lower (P = 0.043) in NY-ESO-1 positive (40.1 ± 7.6 μM) than negative lung cancer cell lines (102.9 ± 14.6 μM). (**C**) A significant negative correlation was observed between NY-ESO-1 methylation and cisplatin sensitivity at 100 μM (Pearson r = 0.597, P = 0.031) and 500 μM (Pearson r = 0.686, P = 0.0097).

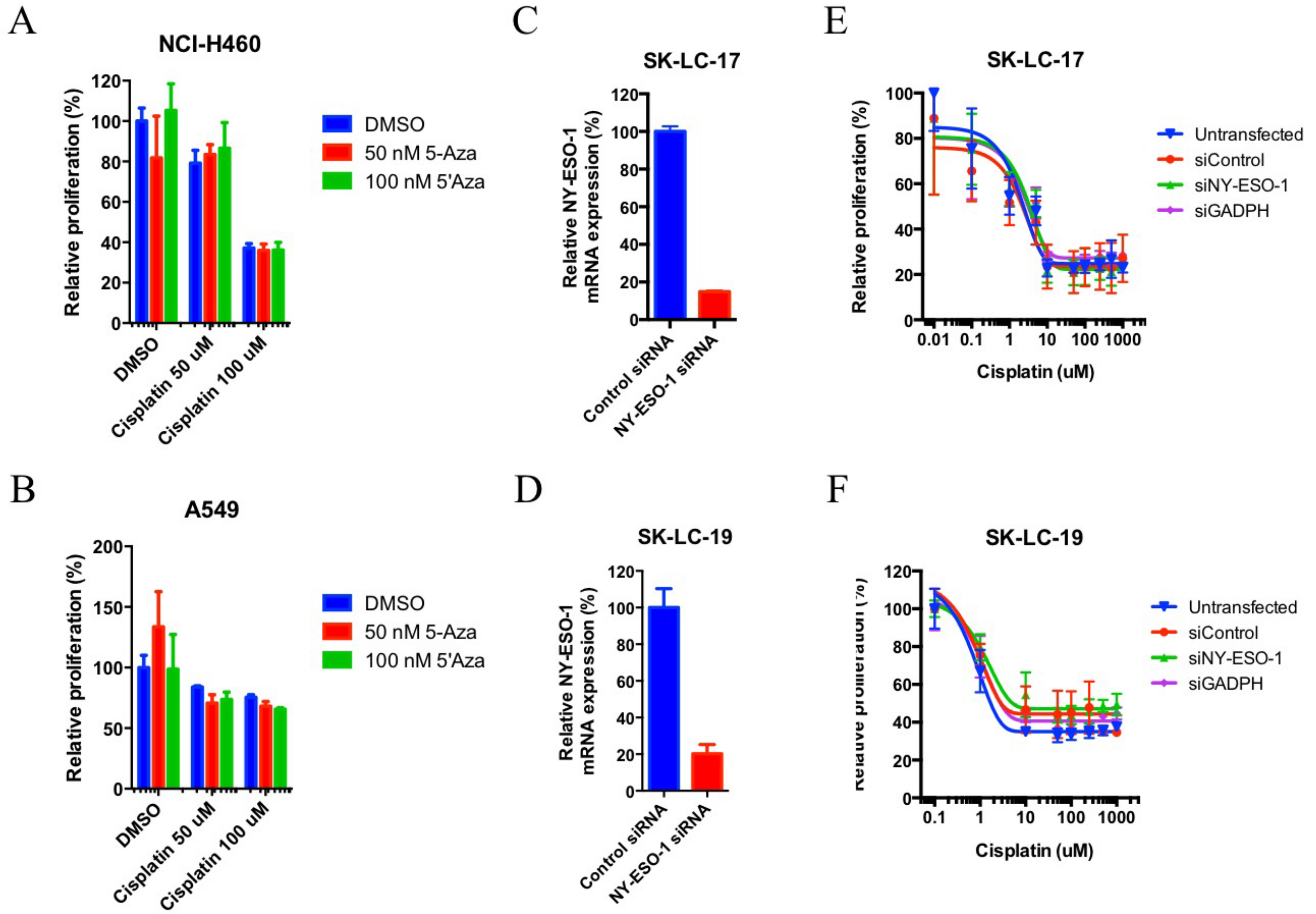

Supplementary Figure 10: Effect of 5-Aza-dC-mediated NY-ESO-1 re-expression and RNAi-mediated knockdown of NY-ESO-1 on cisplatin sensitivity in lung cancer cell lines. (**A–B**) Two hypermethylated lung cancer cell lines, NCI-H460 and A549, were treated with 5-Aza-dC for 72 hr to induced re-expression of NY-ESO-1 at both mRNA and protein level (Supplementary Figure 3 and 5). Cells were re-seeded and followed by cisplatin treatment for 72 hr. No difference in chemotherapy responsiveness after 5-Aza-dC treatment was observed post NY-ESO-1 re-expression. (**C–D**) Quantitative RT-PCR results showing >80% knockdown of NY-ESO-1 in 2 lung cancer cell lines, SK-LC-17 and SK-LC19, using specific NY-ESO-1 targeting siRNA by transient transfection for 48 hr. (**E–F**) Following silencing the NY-ESO-1 gene, no changes of chemosensitivity was observed in. GAPDH siRNA was included as additional control in the experiment. Knockdown of GAPDH was successful, > 80% reduction in mRNA level was observed. Quantitative RT-PCR results for GADPH were not included.

Supplementary Table 1: Cell lines used in this study

Cell Line	Tissue	Disease	Source
**NY-ESO-1 Positive**			
NCI-H2170	Lung	Squamous cell carcinoma	ATCC No. CRL-5928
SK-LC-02	Lung	Adenocarcinoma	MSK
SK-LC-17	Lung	Small cell/anaplastic	MSK
SK-LC-19	Lung	Carcinoma	MSK
LM-MEL-53	Melanoma	Melanoma	LICR
**NY-ESO-1 Negative**			
A549	Lung	Adenocarcinoma	ATCC No. CCL-185
NCI-H460	Lung	Large cell	ATCC No. HTB-177
NCI-H522	Lung	Adenocarcinoma	ATCC No. CRL-5810
NCI-H838	Lung	Adenocarcinoma	ATCC No. CRL-5844
SK-LC-05	Lung	Large cell	MSK
SK-LC-15	Lung	Adenocarcinoma	MSK
HCC827	Lung	Adenocarcinoma	ATCC No. CRL-2868
NCI-H1650	Lung	Adenocarcinoma	ATCC No. CRL-5883
NCI-H1975	Lung	Adenocarcinoma	ATCC No. CRL-5908
NCI-H520	Lung	Squamous cell carcinoma	ATCC No. HTB-182
HCT116	Colorectal	Adenocarcinoma	ATCC No. CCL-247

Abbreviations: ATCC, American Type Culture Collection; MSK, Memorial Sloan Kettering Cancer Center; LICR, Ludwig Institute of Cancer Research.

Supplementary Table 2: MS-PCR and RT-PCR oligonucleotide primers used in this study

Gene	Assay	Type	Primer Sequence (5′ – 3′)
ACTB	MS-PCR	For	5′ TGGTGATGGAGGAGGTTTAGTAAGT 3′
	MS-PCR	Rev	5′ AACCAATAAAACCTACTCCTCCCTTAA 3′
	RT-PCR	For	5′CACCTTCACCGTTCCAGTTT 3′
	RT-PCR	Rev	5′GATGAGATTGGCATGGCTTT 3′
NY-ESO-1 (CTAG1B)	MS-PCR	M_For	5′ CGTAGGGGTAGTAAGGGTTTC 3′
	MS-PCR	M_Rev	5′ ACTCAAACAAACGACTCTCCG 3′
	MS-PCR	UM_For	5′ GTGTAGGGGTAGTAAGGGTTTT 3′
	MS-PCR	UM_Rev	5′ CAACTCAAACAAACAACTCTCCA 3′

Abbreviations: ACTB, beta-actin; NY-ESO-1, New York-Esophageal-1; MS-PCR, methylation-specific polymerase chain reaction; RT-PCR, reverse transcription polymerase chain reaction; For, forward primer; Rev, reverse primer; M_For, forward primer detecting methylation sequence; M_Rev, reverse primer detecting methylation sequence; UM_For, forward primer detecting unmethylated sequence; UM_Rev, reverse primer detecting unmethylated sequence.

Supplementary Table 3: Immunocytochemistry results for NY-ESO-1 and PD-L1 protein expression in lung tumour cell lines

A

	NY-ESO-1 expression		PD-L1 expression	
**Tumour Cell Line**	**Basal, untreated**	**5-Aza-dC treated**	**Basal, untreated**	**5-Aza-dC treated**
A549	Negative	*Positive*	Negative	*A few cells +ve*
HCC827	Negative	Negative	*Positive (Weak)*	*Positive (Strong)*
NCI-H460	Negative	*Positive*	Positive	*Positive*
NCI-H520	Negative	*Positive*	Negative	Negative
NCI-H522	Negative	A few cells +ve	Negative	Negative
NCI-H838	Negative	A few cells +ve	Negative	*Positive*
NCI-H1650	Negative	Negative	Negative	*Positive*
NCI-H1975	Negative	Negative	*A few cells +ve*	*Positive*
NCI-H2170	*Positive*	nd	nd	nd
SK-LC-02	*Positive*	nd	nd	nd
SK-LC-05	Negative	*A few cells +ve*	*Positive (Weak)*	*Positive (Strong)*
SK-LC-15	Negative	Negative	*Positive*	*Positive*
SK-LC-17	*Positive*	nd	nd	nd
SK-LC-19	*Positive*	nd	nd	nd

B

		NY-ESO-1 expression	
		**Negative**	**Positive**
PD-L1 expression	Negative	5	3
	Positive	5	1

P = 0.5804 (Fisher's exact test).

Supplementary Table 4: Univariate analysis to test for association between NY-ESO-1 methylation status and the survival of NSCLC patients in adjuvant chemotherapy treated or untreated sub groups

-> AdjChemo = 0 (No)

Cox regression -- Breslow method for ties

No. of subjects = 50 Number of obs = 50

No. of failures = 42

Time at risk = 1318.032853 LR chi2(1) = 4.37

Log likelihood = −132.37764 Prob > chi2 = 0.0367

<table>
<tr><th>_t |</th><th>Haz. Ratio</th><th>Std. Err.</th><th>z</th><th>P>|z|</th><th>[95% Conf.</th><th>Interval]</th></tr>
<tr><td>NY-ESO-1 Methylation (Low vs High) |</td><td>2.521831</td><td>1.021839</td><td>2.28</td><td>0.022</td><td>1.139755</td><td>5.579822</td></tr>
</table>

–> AdjChemo = 1 (Yes)

Cox regression –– no ties

No. of subjects = 44 Number of obs = 44

No. of failures = 24

Time at risk = 1217.200002

LR chi2(1) = 0.03

Log likelihood = −71.758386 Prob > chi2 = 0.8638

<table>
<tr><th>_t |</th><th>Haz. Ratio</th><th>Std. Err.</th><th>z</th><th>P>|z|</th><th>[95% Conf.</th><th>Interval]</th></tr>
<tr><td>NY-ESO-1 Methylation (Low vs High) |</td><td>1.115184</td><td>.6998912</td><td>0.17</td><td>0.862</td><td>.325935</td><td>3.815592</td></tr>
</table>

Supplementary Table 5: Univariate analysis to test for association between NY-ESO-1 expression and the survival of NSCLC patients in adjuvant chemotherapy treated or untreated sub groups

–> AdjChemo = 0 (No)

Cox regression -- Breslow method for ties

No. of subjects = 50 Number of obs = 50

No. of failures = 42

Time at risk = 1318.032853 LR chi2(1) = 5.06

Log likelihood = −132.03119 Prob > chi2 = 0.0245

<table>
<tr><th>_t |</th><th>Haz. Ratio</th><th>Std. Err.</th><th>z</th><th>P>|z|</th><th>[95% Conf.</th><th>Interval]</th></tr>
<tr><td>NY-ESO-1 Expression (+ve vs –ve) |</td><td>2.155036</td><td>.705268</td><td>2.35</td><td>0.019</td><td>1.134712</td><td>4.092824</td></tr>
</table>

–> AdjChemo = 1 (Yes)

Cox regression –– no ties

No. of subjects = 44 Number of obs = 44

No. of failures = 24

Time at risk = 1217.200002

LR chi2(1) = 0.77

Log likelihood = −71.387196 Prob > chi2 = 0.3797

<table>
<tr><th>_t |</th><th>Haz. Ratio</th><th>Std. Err.</th><th>z</th><th>P>|z|</th><th>[95% Conf.</th><th>Interval]</th></tr>
<tr><td>NY-ESO-1 Expression (+ve vs –ve) |</td><td>.653492</td><td>.3290846</td><td>−0.84</td><td>0.398</td><td>.2435529</td><td>1.753425</td></tr>
</table>

Supplementary Table 6: Multivariate analysis of clinicopathological features associated with survival in patients

Cox regression -- Breslow method for ties						
No. of subjects	=	94	Number of obs =		94	
No. of failures	=	66				
Time at risk	=	2535.232855	LR chi2(1)	=	16.89	
Log likelihood	=	−243.09972	Prob > chi2	=	0.0097	

_t	Haz. Ratio	Std. Err.	z	P>|z|	[95% Conf.	Interval]
TNM Staging (IIIB vs IIIA)	1.082017	.5232458	0.16	0.871	.4193793	2.791652
Histology (SQ vs ADC)	1.370266	.584912	0.74	0.461	.5935547	3.163362
Histology (Others vs ADC)	1.386865	.5401777	0.84	0.401	.6463903	2.975592
AdjChemo (Yes vs No)	.6912318	.2093874	−1.22	0.223	.3817495	1.25161
NY-ESO-1 Methylation (Low vs High)	3.588361	1.527267	3.00	0.003	1.558154	8.263839
NY-ESO-1 Methylation (Low): AdjChemo (Yes)	.210812	.164579	−1.99	0.046	.0456426	.9736894

SQ, squamous cell carcinoma; ADC, adenocarcinoma; AdjChemo, adjuvant chemotherapy.
